# Editorial

**Published:** 1860-03

**Authors:** 


### EDITORIAL.

### IMPROVEMENTS IN MEDICAL COLLEGE INSTRUCTION. OPINIONS OF THE MEDICAL PRESS, ETC.

Improvements in the general system of Medical College Instruction, have constituted the subject of discussion, or of Editorial comment in many of the Medical Journals during the last few months. Some of these discussions have had reference to the next meeting of the *American Medical Association*, and the convention of delegates from the Medical Colleges to be held in New Haven, on the first Monday and Tuesday in June next; and some have had reference to the organization of the Medical Department of Lind University in this city. The plan adopted by the latter Institution has been noticed at some length, and in general terms of approval, by the *Boston Medical and Surgical Journal*, the *New York Medical Gazette*, the *Medical and Surgical Reporter*, of Philadelphia, and the *Cincinnati Lancet and Observer.*

The *New Orleans Medical News and Hospital Gazette* warmly sanctions the plan, as a move in the right direction, and compliments the Faculty for their boldness in adopting it, but expresses the fear that the change is *too great* to meet with a ready support from the profession. The *Nashville Journal*, without directly approving or disapproving, expresses the opinion that the tendency of the professional mind is decidedly towards the establishment of "one solid term of nine months," similar to the arrangement adopted by the University of Virginia. The *Peninsular and Independent* of Detroit, commends the plan as a great improvement over that of the Rush Medical College, but still not sufficient to meet the wants of the profession. Prof. A. B. Palmer, one of the Editors of that Journal, expresses the opinion that the student should be required to study four years, and attend two full college terms of nine months each. He alludes to our having formerly proposed an annual college term of *nine* months duration, and intimates an inconsistency in having now assented to the adoption of one of only *five* months. It is true that two years since we proposed a plan of Medical College Instruction, which would make the annual College term embrace *nine* months, to be divided into a junior department of *four* months for first course students, and a senior department of *five* months for second course students. Further reflection, however, satisfied us that *four* months was too short a period for the junior course. Hence, when an opportunity presented for choosing a plan for actual adoption in practice, we preferred to increase the number of Professors, so that both departments might be carried on simultaneously, and make each full *five* months; which would be just equal to a single or consecutive term of *ten* months.

Prof. Palmer will thus see that instead of consenting to the dropping off of *four* months from our former plan for "prudential reasons," we have insisted on the actual addition of *one* month to the aggregate time of College instruction. The only serious objection that we have seen or heard urged against the plan of the Lind University, is, that although it requires the student to attend two full courses of lectures, equal in duration to the courses in any of our best Colleges, yet it requires but one course in each department. In other words, its courses are not repetitional; and it is said that one course on any given subject is not sufficient to fully impress its facts on the mind of the student. In proof of this, Prof. Palmer cites the fact that students almost always think their second course is far more profitable to them than their first. This objection as applied to the system we advocate, though plausible, is nevertheless fallacious. It is undoubtedly true, that under the prevalent system of College instruction, by which the student is required to listen to lectures on all the branches promiscuously each term, his second course is far more profitable than his first; and for a very obvious reason. Almost all students during their first year of study, properly confine their reading to Anatomy, Physiology, and Materia Medica. Hence, they come to their first course of lectures with no knowledge of the other branches. But their minds are immediately crowded with from five to six lectures per day, embracing not only a review of those branches with which they have acquired some degree of familiarity by previous study, but also four or five others to which they are entire strangers.

The number of topics thus crowded upon their attention daily, with the desire to spend the interval between the lecture hours in the dissecting room, keeps their minds in a state of confusion, which even the best students do not recover from until the term is more than half gone. Thus they actually loose about half the value of their first course by the absence of a due preparation to understand it, and the want of time to review and reflect upon it during its progress. Hence, it is not surprising that under such a system almost every student should feel conscious of obtaining far more satisfaction from his second, than from his first course. But suppose, that instead of all this, the student coming to his first course should have his attention confined exclusively to descriptive and practical Anatomy, Inorganic Chemistry, Physiology, Materia Medica, and General Pathology; and that he should receive as many lectures on these subjects alone as is usually devoted to all the branches. Suppose further, that the term should be sufficiently extended to require only four lectures to be given daily, thereby allowing ample time to digest them, and attend to extra lessons in Practical Anatomy and the use of the microscope, would he feel any of that embarrassment and confusion which necessarily pertained to the other course? On the contrary, would he not necessarily receive and treasure up the facts and principles embraced in these particular branches far more fully and perfectly than he could by even two courses on them, given in a much shorter time, and mingled promiscuously with courses on all the other branches at the same time?

Let the reader reflect on this seriously. By the system we advocate, the junior students get 480 lectures on the five branches just named, in twenty weeks; making an average of 96 lectures for each branch, besides extra lessons with the Microscope, Dissections, and daily examinations.

While first course students in the Rush Medical College, or any other on the old plan, get 462 lectures in sixteen weeks, on seven or eight different branches; making at most, an average of only 66 lectures on each branch.

Can any reflecting man fail to see how much more extensively and thoroughly each branch in our junior department would be presented to the class, than the same branches could be in a course on the ordinary plan?

Again, by the ordinary college course, the student attending his second term, gets a repetition of the same 462 lectures to which he listened during his first term, and in the same heterogenious order. While by our plan, the student coming to his second or senior term, having had his attention during the first term limited exclusively to a full and protracted course on the five most elementary branches, now brings to the review of the six remaining branches a mind fully prepared and disciplined for the work. His previously acquired familiarity with the elementary departments, enables him to fully appreciate every fact and principle presented in the lectures on the more practical branches; while the privilege of limiting his attention exclusively to these during his senior course greatly facilitates his progress. With these advantages, the senior student finds no difficulty during the five months embraced in our senior term, in thoroughly digesting the 480 new lectures on Surgical Anatomy, Organic Chemistry, Principles and Practice of Medicine, Principles and Practice of Surgery, Obstetrics and Diseases of Women and Children, and Medical Jurisprudence; with the addition of 160 Medical and Surgical Cliniques given at the bed-side of the sick, which together constitute our senior course. To convey a just idea of the difference between the ordinary plan of college instruction pursued in this country, and that advocated and adopted by us, we must pursue the contrast further.

In the ordinary system, the entire collegiate instruction of the student embraces but *seven* regular courses of lectures, aside from cliniques, on the same number of branches of medical science; while in the system we adopt, it embraces *eleven* regular courses, on as many different branches. It is well known that the important branches of Organic Chemistry,

Histology, Surgical Anatomy, and Medical Jurisprudence, though enumerated in many of the catalogues and annual announcements, are practically omitted from the ordinary college courses, simply because they are severally appended to other branches which absorb all the attention of the lecturer through the short term of sixteen weeks. The large number of Professorships in our system, secures to these important branches as full a consideration as to any of the rest.

With this comparison, showing so clearly the more methodical and natural arrangement of studies; the greater number of branches included in the course; the much larger number of lectures devoted to each branch; and the increased length of the terms, by which ample time is secured for Dissections, Microscopic Demo strations, and Hospital clinical attendance; we might well claim that these advantages possessed by our system, far more than counterbalance any good to be derived from a mere *repetition* of the same courses. But in addition to all these advantages, we claim that our system secures to the student a far more desirable and profitable repetition of facts and principles involved in the different departments of medicine than the ordinary system. In Anatomy, for example, by the ordinary system, the Professor commences with Osteology, and progresses through the demonstration of bones, ligaments, muscles, blood vessels, nerves, and viscera, and in 49 cases out 50, the end of the college term comes before he has completed the consideration of these. In his next course he commences at the same place, and goes over the same field again. Hence, no matter how many courses the student may attend, he is never carried beyond the elementary demonstrations in descriptive Anatomy.

By our system, however, he gets in his first course a more protracted and complete demonstration of descriptive Anatomy from the Professor in that department; and in his second, he has all the important facts of his first course brought under review, and their relations to surgery demonstrated by the Professor of Surgical Anatomy, and operations of surgery.

So with the subject of Chemistry. By the ordinary system the Professor of Chemistry begins each annual term with the elements of Inorganic Chemistry, and in more than 49 cases out of 50, he reaches the end of the term before he has passed over more than two-thirds of the field embraced in that department of the science alone.

Prof. Blaney, of Rush Medical College, who certainly ranks among the best teachers of Chemistry in our country, generally finds himself just in the midst of a consideration of the *metals*, when the last week of the college term comes. He then, to avoid the charge of entirely omitting Organic Chemistry and Toxicology, generally devotes from two to four lectures to these important topics. The student attending his second course, simply passes over the same part of the science again. By our plan the junior student gets 60 lectures on Elementary and Inorganic Chemistry, and in his senior course he gets 60 more on Organic Chemistry, by which the facts and principles of the inorganic course are necessarily brought under review in their application to the study of organic substances. It is thus easy to perceive that while our system of Medical College Instruction does not consist in an endless repetition of courses on the same part of each branch, it does permit such a repetition of the facts and principles taught in the one department, as will bring them fully under review in the other, and show their direct application to the elucidation of the several branches taught in the senior department. And this we regard as just the kind of repetition that the student most needs.

We are confident, both from careful reflection, and from the experience of the present session, that the more our system is examined, the better its actual scope and designs are understood, the more certainly will it meet the approval of the profession. In the language of a private correspondent, "if successfully carried out, it will work a great and most decidedly favorable change in our educational system." It is so radically different from the ordinary system of Medical College organization in this country, that many of our friends who cordially approve of both its principles and details, express the fear that it is undertaking too great a change at once; and our friendly New Orleans contemporary reminds us of the old maxim "*Festina Lente.*"

In reply, we must remind our friends that there are some things that cannot be done either slowly or gradually. For instance, we cannot adopt a *principle* of action gradually. The system of Medical College Instruction which we have devised, has for its fundamental principle the division of the annual college term into two departments, with one half of the field of medical science and art assigned to one department, and the other half to the other; by which a strictly methodical system of study is secured to the student, and the aggregate extent of the field of medical college instruction greatly enlarged. No institution can adopt this principle slowly. Each college undertaking to act on the subject, must either adopt it at once by dividing the term, or reject it by adhering to the ordinary plan. Those who control the Medical Department of Lind University have chosen to adopt it, and we are confident that the profession of the North-West will both approve and sustain their choice. We are glad to see indications that the Committees of Conference appointed by the American Medical Association at its meeting in Louisville, and the Convention of Delegates from the Medical Colleges, are giving serious attention to the subject referred to them. We hope they will mature such measures as will meet the approval of both these bodies at their next meetings, and will speedily result in a great improvement of the ordinary plan of College instruction throughout the whole country.

---

### PUBLIC ANNUAL COMMENCEMENT IN THE MEDICAL DEPARTMENT OF LIND UNIVERSITY.

The first public commencement in the Medical Department of Lind University, will be held in the Second Presbyterian Church, on the corner of Wabash Avenue and Washington Streets, on Monday Evening, March 5th. The exercises will consist of a prayer by the Chaplin, the awarding of a present to the author of the best inaugural thesis; a valedictory address on behalf of the students by one of their number; the confering of the degree of M. D. on those who have passed their examinations satisfactorily; and a valedictory address to the graduates by the President of the Faculty, Prof. H. A. Johnson; after which an entertainment will be given to the graduates and students at the residence of Prof. N. S. Davis. The following candidates having complied with the requisitions of this department of the University, and sustained a satisfactory examination on all the branches taught in the junior and senior courses, will receive the degree of Doctor of Medicine, viz:

C. DeHaven Jones, of Evanston, Illinois.
John Conant, of Rockford, Illinois.
J. S. Jewell, of Attila, Illinois.
Rufus Coggswell, of Illinois.
J. M. Kendall, of Wabash, Indiana.
Lucian Ashley, of Magnolia, Illinois.
Thomas J. Rigg, of Chicago, Illinois.
Horace B. Pike, of Chicago, Illinois.
J. F. Hopkins, of Milwaukee, Wisconsin.
A. D. Andrews, of River Falls, Wisconsin.

The *ad eundem* degree will also be conferred on Drs. Edward C. Dickinson, and Ezra A. Steele, both of this city.

---

### ANNUAL MEETING OF THE ILLINOIS STATE MEDICAL SOCIETY.

The tenth annual meeting of this Society will be held in Paris, Edgar Co., on the 2nd Tuesday in *May* next. We hope the profession throughout the State will remember the time, and that a full representation from all sections will be present. The people of Paris will give them a most hospitable reception. The following preamble and resolutions offered by Dr. T. D. Washburn at the last meeting, will come up for consideration during the coming session. They have been published in the Cincinnati *Lancet and Observer*, and in the *North American Medico Chirurgical Review*, and the sentiments they contain were fully endorsed by the editors of both those Journals.

We call particular attention to these resolutions at this time, because our neighbor of the *Chicago Medical Journal* has two or three times alluded to them as proposing action disrespectful to the profession of New Haven, where the American Medical Association is to hold its next meeting, and at the same time he has carefully abstained from publishing the resolutions so that his readers might judge for themselves. Such a course is not only contemptible in itself, but manifestly unjust to the members of our own State Society. The preamble and resolutions are as follows:

*Whereas*, the American Medical Association is a national Association, composed of delegates and members from all parts of the United States, meeting on terms of perfect equality:

Therefore, *Resolved*, That in the opinion of this Society, all the officers of the Association should be selected strictly with reference to *merit*, and without any regard to their place of residence.

*Resolved*, That the custom of selecting the President of the Association exclusively from the Profession of the City in which the Annual Meeting is held, is not only derogatory to the *general* character of the organization, and calculated greatly to lessen the honor which should attach to that office, but past experience has shown that it leads directly to local divisions, jealousies, and injurious partisan strife.

*Resolved*, That the delegates from this Society to the Association, be instructed to use their influence to abrogate the custom alluded to in the preceding resolution.

*Resolved*, That the secretary be directed to furnish copies of the foregoing resolutions to other state and local Medical Societies, and ask their attention to the same.

---

### ANNUAL COMMENCEMENT OF RUSH MEDICAL COLLEGE.

The Public commencement of Rush Medical College was held in the College, on the evening of the 15th of February. The lecture room was well filled by an attentive audience. The exercises consisted simply in prayer by the chaplain, the conferring of the degree of Doctor of Medicine on 34 candidates, and the reading of a valedictory address by the President of the College, Prof. D. Brainard. The address commenced with a brief allusion to the past history of the College, and then proceeded to discuss the "wants of the American Physician," and to hold up Hunter and McClellan as pre-eminently worthy of imitation by all members of our profession. The whole was, suitably *seasoned* with non-complimentary allusions to the "*pseudo-reformers* of the *present day*." The whole number attending that College, during the past session, is reported to be about 100, being a moderate diminution from the previous session, and a less number than has attended during any previous year since 1849.

---

### SUMMER COURSE OF MEDICAL INSTRUCTION IN CONNECTION WITH MEDICAL DEPARTMENT OF LIND UNIVERSITY.

Immediately after the close of the regular annual course of instruction in this Institution, which occurs on the first Monday in March, a summer course of instruction will be commenced. This course will embrace instruction in Clinical Medicine in the Mercy Hospital and the Chicago City Dispensary, by Prof. N. S. Davis; in Clinical Surgery, by Prof. E. Andrews, in the same Institutions; in the more important topics connected with Midwifery and Diseases of Women, by Prof. W. H. Byford; in Analytical Chemistry, by Prof. F. Mahla; in Practical Anatomy, by Dr. H. Wardner; in Histology and Microscopy, by Prof. J. H. Hollister; on Auscultation and Percussion, by Dr. E. A. Steele; and on the Diseases of the Genito Urinary Organs, by Dr. S. C. Blake.

If Prof. Deville remains in the city, he will also give a course of instruction in Surgical Anatomy and Operative Surgery.

The instruction in all the above named departments will be FREE for the attendance of any regular student of medicine, except those of Analytical Chemistry, Practical Anatomy, and Surgical Anatomy with Operative Surgery. For each of these a moderate fee will be charged to defray the expense of material used, etc. To those who wish to pursue the study of medicine in Chicago the coming summer, this will afford every needful facility.

---

### A QUESTION OF ETHICS.

"1. *Reductions of Dislocations of the Hip Joint by Manipulation.*

Since making the note published in our issue for October, a case of this accident has fallen under our notice, which differs in some respects from those heretofore met with. It was that of Page, the baggage-man, injured at the accident on the Chicago and Northwestern R. R., near Watertown, Wis. The head of the bone lay in the ischiatic notch. Near three days had elapsed since the accident. Efforts at reduction had been made by a surgeon from Milwaukee, who, after applying, as we were informed, a very great mechanical power, without success, pronounced it a *fracture of the neck of the femur*, but left it without applying any dressing. The ligaments of the hip joint appeared to be entirely torn across and much contusion produced, as the member could be moved more freely than is usual in such cases.

Reduction by Reid's method was effected, but less readily than is usual. After the reduction, a slight tetanic spasm supervened, which lasted several days, but which, at our last visit, Nov. 24, three weeks after the accident, had almost entirely disappeared.

We have felt called upon in this case, contrary to uniform practice, to make a statement of facts, which may seem to reflect upon the surgeon previously called, in consequence of the attacks repeatedly made upon us in the *Milwaukee Daily News*."

We copy the above from the 12th number of the *Chicago Medical Journal*, because it contains a grave charge against one of the most upright and distinguished surgeons in the neighboring State of Wisconsin. The paragraph not only charges the "Surgeon from Milwaukee" with ignorance in having mistaken a dislocation for a fracture of the neck of the femur, and with having used undue force or violence in efforts to reduce the limb; but also in a marginal note, he is indirectly accused of having assailed Dr. Brainard through the columns of a Milwaukee daily paper. As a just and proper offset to these charges, we publish the following statement, communicated by the surgeon referred to by Dr. Brainard.

Milwaukee, Jan., 1860.

E. A. Steele, M. D.,

Dear Sir:—

I was at Watertown soon after the "Belleville Calamity." Saw Mr. Paige, and will cheerfully give you the *facts* as far as I know them. I found him with a dislocation of the left shoulder, and injury of the corresponding hip. After a reduction of the shoulder, I examined the hip; it was not an easy matter to determine the true nature of the injury. The position of the limb corresponded very nearly to a dislocation into the ischiatic notch; but on moving it, doubt was at once thrown upon that supposition, by the ease and latitude of abduction, as well as the readiness with which moderate extension would bring the injured limb to correspond in length with the sound one. Thus far, no other surgeon, so far as I know, had examined Mr. Page. I foresaw difficulties in the case, and left it until the delegation from Milwaukee arrived, which was soon after. A more thorough examination was then instituted, resulting in more or less difference of opinion—some regarding it as a dislocation—others a fracture within the capsular ligament, or of the acetabulum. It was, however, agreed that an effort at reduction should be made, which if successful, would settle the case; if not, still might, by bringing the fractured pieces in contact, thereby producing crepitation. During the attempt to reduce, I thought, and still believe, I felt several times distinct crepitation. This, of course, settled the question in my mind that a fracture of some kind existed. A diversity of opinion still existed, and several other attempts were made to reduce; during which a number of the medical gentlemen declared they also detected crepitus.

I returned to Milwaukee the next day. Dr. Brainard saw Page the same day in the afternoon, and said he reduced the dislocation, and that in a few weeks he (Page) would be well.

I returned to Watertown the next day after. Saw Page and some of his friends; they, of course, were feeling very well, for I had told them Page would never entirely recover. On this occasion I reiterated the same opinion, but added the hope that I might some day be disappointed, by learning that he had *fully* recovered.

Through the courtesy of a medical friend (I do not take the *Journal*) I have seen this evening, for the first time, the Editor's remarks on "reduction of dislocation of the hip joint by manipulation."

He states, that he was informed, that a surgeon from Milwaukee applied great mechanical power, tearing entirely across the ligaments of the hip joint, producing much contusion, etc. He also says, "the reduction by Reid's method was effected, but less readily than is usual."

Was the ruptured state of the ligaments the cause of the unusual difficulty? Whoever informed Dr. Brainard as to the force used, at least when I was present, was either ignorant or malicious. The statement is unqualifiedly false, and can be so proved by every medical gentleman present on the occasion, and they were quite numerous. Further, he speaks of *slight* tetanic spasms supervening after the reduction, lasting *several* days. It is well known, that for many weeks he was in a most critical condition, and at times his life even was despaired of by both friends and medical attendants.

I have never taken the *Milwaukee Daily News*, and should never have seen the "attacks" made on the Doctor had they not been shown me by others. I will send you, if I can find them, the paper that you may see how badly the Doctor was "attacked."

Very Respectfully,
Your Obt. Servant,
E. B. WOLCOTT.

We have further learned from a reliable source, that no post mortem examination was made; but that after suffering for a considerable time after the operation for reducing the dislocation of the hip, by Dr. Brainard, from tetanic muscular contractions of considerable severity, he improved slowly until he was removed to McHenry, where he was attacked with vomiting and other symptoms of gastritis, which persisted until fatal exhaustion supervened.

---

### PROFESSIONS AGAINST PRACTICE.

In the *Chicago Medical Journal* for June last, among other misrepresentations, the editor stated that the ordinary course of instruction in the Rush Medical College embraced an aggregate of 570 lectures, while a course in the Medical department of Lind University embraced only 430; and therefore the founders of the latter had narrowed instead of extended the field of College instruction. Since then each of these institutions have actually completed their annual course of lectures. The regular lecture term in the Rush Medical College continued just fifteen weeks and two days. Six lectures per day were given except on Wednesdays and Saturdays when only three were given, the afternoon of those days being devoted to clinical purposes. This gave thirty lectures per week for fifteen weeks and twelve lectures for the two additional days, making an aggregate for the whole term of just 462 lectures instead of the 570 previously claimed.

The annual term in the Medical department of Lind University is five months or twenty full weeks. The term consists of two departments, the Junior and Senior. The first embraces four lectures per day throughout the whole term on Anatomy, Physiology, and Histology, Materia Medica, Inorganic Chemistry, and General Pathology; making an aggregate of 480 lectures. Hence a first course student in this school hears eighteen more lectures on these five branches, than are given in the Rush Medical College on all the departments of medical science. While a student attending his second course in the latter institution hears a *repetition* of the same 462 lectures. In the University he enters the Senior department and listens to 480 more lectures on Organic Chemistry, Surgical Anatomy, Practical Medicine, Practical Surgery, Obstetrics, and Medical Jurisprudence.

The clinical advantages of the two schools during the terms just closed were as follows: In the Rush Medical College each student taking the Hospital tickets, was admitted to 3 Hospital and 1 College Clinique each week, making an aggregate of 60 Cliniques for the term. In the University School, each student taking the Hospital ticket was admitted to 6 Hospital and 2 College Cliniques per week, making an aggregate of 160 Cliniques during the term. Thus it will be seen that a student depending on Rush Medical College for education, would get 462 lectures and 60 cliniques once repeated. While in the Medical Department of Lind University, he would get 960 consecutive lectures, and 160 cliniques.

And this is what the senior editor of the *Chicago Medical Journal* called *narrowing the field of College Instruction!*

---

*The American Medical Association* will hold its thirteenth annual meeting at New Haven, on the *first Tuesday of June*, 1860. The secretaries of local societies, colleges and hospitals, are requested to forward to the undersigned, the name of delegates as soon as they are appointed.

STEPHEN G. HUBBARD, M. D., *Secretary*,
*New Haven, Ct.*

